# Paeoniflorin Inhibits Mesangial Cell Proliferation and Inflammatory Response in Rats With Mesangial Proliferative Glomerulonephritis Through PI3K/AKT/GSK-3β Pathway

**DOI:** 10.3389/fphar.2019.00978

**Published:** 2019-09-09

**Authors:** Bihao Liu, Jin Lin, Lixia Bai, Yuan Zhou, Ruirui Lu, Peichun Zhang, Dandan Chen, Honglian Li, Jianping Song, Xusheng Liu, Yifan Wu, Junbiao Wu, Chunling Liang, Jiuyao Zhou

**Affiliations:** ^1^Department of Pharmacology, School of Pharmaceutical Sciences, Guangzhou University of Chinese Medicine, Guangzhou, China; ^2^College of Chinese Materia Medica, Guangdong Food and Drug Vocational College, Guangzhou, China; ^3^Science and Technology Industrial Park, Guangzhou University of Chinese Medicine, Guangzhou, China; ^4^The Second Affiliated Hospital, Guangzhou University of Chinese Medicine, Guangzhou, China

**Keywords:** mesangial proliferative glomerulonephritis, paeoniflorin, mesangial cells proliferation, inflammatory response, PI3K/AKT/GSK-3β pathway

## Abstract

Mesangial proliferative glomerulonephritis (MPGN) is the most common type of chronic kidney disease in China, characterized by mesangial cell proliferation and inflammatory response. Paeoniflorin, an effective composition extracted from *Radix Paeoniae* Alba, has been used for various kinds of kidney diseases. However, there are no studies reporting the effects of paeoniflorin on MPGN. The present study aims to investigate whether paeoniflorin plays a role in MPGN and confirm the underlying molecular mechanisms. Our results manifested that paeoniflorin strongly restrained 24 h urinary protein and promoted renal function and dyslipidemia in a MPGN rat model. Moreover, paeoniflorin attenuated mesangial cell proliferation and inflammation both in MPGN rats and human mesangial cells (HMCs) treated with lipopolysaccharide (LPS). In detail, paeoniflorin decreased the number of mesangial cells and expressions of proliferation marker Ki67 in MPGN rats. Paeoniflorin also inhibited HMC proliferation and blocked cell cycle progression. In addition, the contents of inflammatory factors and the expressions of macrophage marker iNOS were decreased after paeoniflorin treatment. Furthermore, we found that the protective effect of paeoniflorin was accompanied by a strong inhibition of the phosphatidylinositol 3-kinase (PI3K)/AKT/glycogen synthase kinase (GSK)-3β pathway. Paeoniflorin enhanced the inhibitory effect of PI3K inhibitor LY294002 and suppressed the activated effect of PI3K agonist insulin-like growth factor 1 (IGF-1) on PI3K/AKT/GSK-3β pathway. In conclusion, these results demonstrated that paeoniflorin ameliorates MPGN by inhibiting mesangial cell proliferation and inflammatory response through the PI3K/AKT/GSK-3β pathway.

## Introduction

Mesangial proliferative glomerulonephritis (MPGN) is defined by pathological injury pattern with increased cell number and extracellular matrix in the mesangial area (Qin et al., 2013; Yang et al., 2018). Abnormally proliferative mesangial cells would release inflammation mediators, which often leads to interstitial fibrosis and glomerulosclerosis, resulting in irreversible progressive glomerulosclerosis, eventually turning into end-stage renal disease (ESRD) (Geng et al., 2016). Proliferation and inflammation of mesangial cells plays a crucial role in the progression of MPGN. However, the underlying mechanism of MPGN is still unknown, and there is no effective therapeutic drug to treat MPGN (Bai et al., 2017). Therefore, it is of vital importance to explore the pathogenesis of MPGN and search for effective drugs.

Phosphoinositide-3-kinase (PI3K) is a significant regulator of various signal transduction pathways, which mainly controls cell growth, apoptosis and metabolism (Zhang et al., 2014; Fruman et al., 2017). The serine/threonine kinase (AKT), a downstream kinase of PI3K, plays an important role in cell death and survival (Li et al., 2018). After receptor stimulation, AKT targets numbers of substrates for phosphorylation. One of the major effectors downstream of AKT is glycogen synthase kinase (GSK)-3β, by initiating phosphorylation at its serine 9 residue (Wang et al., 2014). Research proved that the activation of the PI3K/AKT/GSK-3β signaling pathway is vital for cell proliferation (Wang et al., 2012). Our previous study verified that the AKT/GSK-3β pathway is activated when mesangial cells proliferate, and it could be an efficient path to suppress abnormal proliferation and inflammation response through down-regulating the AKT/GSK-3β pathway (Wu et al., 2018).

Paeoniflorin is an active ingredient isolated from of *Radix Paeoniae* Alba, the dried root of *Paeonia lactiflora* Pallas which has been used in traditional Chinese medicine for many years. Paeoniflorin exhibits numerous pharmacological effects, such as anti-inflammation, immunomodulation, antioxidation and anti-proliferation of cancer cells (Gu et al., 2016; Zhai et al., 2016; Song et al., 2017; Zhang et al., 2018). Recent research and our previous study found that paeoniflorin improved some experimental models of kidney disease, including diabetic nephropathy (Zhang et al., 2017), nephrotic syndrome (Lu et al., 2017), acute renal injury (Wang et al., 2016) and renal fibrosis (Zeng et al., 2013). More importantly, paeoniflorin ameliorated advanced glycation end product (AGE)-induced mesangial cell injury partly by anti-inflammation (Zhang et al., 2013; Chen et al., 2017). In recent research, paeoniflorin played a neuroprotective effect through inhibiting the calpain/AKT/GSK-3β pathway in SH-SY5Y cells (Ma et al., 2018). Meanwhile, some studies have shown that paeoniflorin promoted phosphorylation of AKT and GSK-3β in HepG2 cells and diabetic rats (Ma et al., 2017; Sun et al., 2017). Therefore, it is unclear whether paeoniflorin could protect MPGN, as well as the underlying mechanism between them. The present study aims to investigate the effects of paeoniflorin on MPGN *in vivo* and *in vitro* and explore the molecular mechanism related to the PI3K/AKT/GSK-3β pathway.

## Materials and Methods

### Animal Treatment

Fifty male Sprague Dawley rats weighting 180-220 g were provided by Guangzhou University of Chinese Medicine Research Center for Experimental Animal (Certificate NO. SCXK 2013-0020). All the rats were housed in an air-conditioned room at 25 ± 2 °C and 65% humidity with a 12h light/12h dark cycle in specific pathogen-free conditions (Environment certificate NO. 2013-0085).

The MPGN rat model was established under an improvement of Hogendoorn’s method (Hogendoorn et al., 1990). Except for 8 rats in the normal group, the other rats underwent left nephrectomy after being anesthetized with 35 mg/kg pentobarbital sodium by intraperitoneal injection. One week later, all the model rats were subcutaneously injected with 3 mg bovine serum albumin (BSA, Sigma-Aldrich, St. Louis, MO, USA) emulsified in 0.1 ml Freund’s complete adjuvant (Sigma-Aldrich, St. Louis, MO, USA) twice in two weeks. At the end of the third week, the rats were intraperitoneally injected with 0.5, 1.0, 1.5 and 3 mg BSA every 1 hour respectively. Afterwards, every rat was injected with BSA in the tail vein from 0.5 mg to 3.0 mg every other day for 11 days with an increment of 0.5 mg; then the increment became 0.5 mg per week until the ninth week. In the interval time between tail vein injection, every rat was given BSA by intraperitoneal injection twice the dose of that with tail vein injection. At the end of the fifth week, every rat was injected with 100 μg lipopolysaccharide (LPS, Sigma-Aldrich, MO, USA) in the tail vein. At the same time, 24 h urinary protein of all rats was detected using Coomassie brilliant blue method (CoWin Biosciences, Beijing, China). Rats with higher urinary protein than the normal rats were randomly divided into four groups (each with 8 rats): model group, prednisone group (5 mg/kg, Guangdong Huanan Pharmaceutical, China), high dose group and low dose group of paeoniflorin (100 mg/kg, 50 mg/kg, Chengdu Herbpurify, China). The dose of paeoniflorin was decided according to our previous study (Lu et al., 2017). Prednisone was regarded as a positive drug, which is a common drug in clinic to treat kinds of chronic kidney diseases. Drugs were orally administered to rats daily from the sixth week to the tenth weeks.

All animal experimental programs were in accordance with the guidelines of the Animal Ethics Committee of Guangzhou University of Chinese Medicine submitting to the guidelines of the European Community and the National Institute of Health of the USA. All efforts were made to minimize suffering during animal experiments.

### Measurement of 24 h Urinary Protein and Biochemical Parameters

Metabolic cages were used to collect 24h urine samples weekly to detect urinary protein from the fifth week. During the urine collection period, rats were fasting and given free access to water. On the last day of animal experiment, animals were euthanized by intraperitoneal injection with pentobarbital sodium. Blood from abdominal aorta was collected and then centrifuged at 3000 rpm for 10 min at 4°C to acquire serum for biochemical analysis. The level of serum creatinine (SCr), blood urea nitrogen (BUN), total cholesterol (TC) and triglycerides (TG) were measured according to the manufacturer’s instructions (Nanjing Jiancheng Bioengineering Institute, China).

### Histological Analysis

Kidneys of each rat were divided into two parts; one was fixed in 4% paraformaldehyde for histology staining and immunohistochemistry while the rest were frozen at -80°C for western blot analysis. Kidneys fixed for 48h were dehydrated in ethanol and embedded in paraffin. Renal tissue sections (5-μm thick) were cut and dewaxed by gradient series of ethanol, then stained with hematoxylin-eosin (HE) or periodic acid-Schiff (PAS). The mesangial matrix index (MMI) of PAS stained sections were assessed by Image-Pro Plus 6.0 software, representing the ratio of the mesangial matrix area and glomerulus area. Twenty glomeruli were assessed in each rat kidney.

### Immunohistochemistry

For immunohistochemistry, the dewaxed sections were boiled with sodium citrate to retrieve antigen and soaked in 3% hydrogen peroxide to quench endogenous peroxidase, then blocked with normal goat serum for 30min at 37°C. After that, the sections were incubated with Ki67 (ab15580, Abcam, Cambridge, UK) at 1:500 dilution or iNOS (ab3525, Abcam, Cambridge, UK) at 1:500 dilution overnight at 4°C. After washing with PBS, the sections were incubated with secondary antibody (CoWin Biosciences, Beijing, China) for 30min at room temperature. Subsequently, the sections were counterstained with diaminobenzidine (DAB) and hematoxylin, respectively. All the images were captured with 40× objective under light microscope (TE2000, Nikon, Tokyo, Japan). The number of Ki67 positive cells in the glomerulus of 10 randomly selected nonoverlapping fields was evaluated (n = 4). The integral optical density (IOD) of iNOS of each group (n = 4) was measured by Image-Pro Plus 6.0 software.

### CBA Analysis of Inflammatory Factors

Inflammatory factors in serum of every rat were detected as previously described (Wu et al., 2018). Briefly, the levels of IL-1α, IL-2, IL-10 and IFN-γ were measured with a cytometric bead array kit (Becton Dickinson, SanJose, USA) by FACS Canto II (Becton Dickinson, SanJose, USA) according to the manufacturer’s instruction.

### Cell Culture and Treatment

Human mesangial cells (HMCs) at the fifth passage were purchased from the Advanced Research Center of Central South University (Wuhan, China). HMCs were cultured in RPMI 1640 (Gibco, Grand Island, NY), supplemented with 10% fetal bovine serum (FBS, Biological Industries, Israel), as well as 100 U/ml penicillin and 100 mg/ml streptomycin (Gibco, Grand Island, NY) in a cell incubator with 5% CO_2_ at 37°C.

HMCs were treated with 30 μg/ml LPS (Sigma-Aldrich, MO, USA) for 24 h with or without paeoniflorin at 5, 10, 20 and 40 μM for MTT, Edu, cell cycle, RT-PCR and western blot assays. The dose of paeoniflorin for the *in vitro* experiment was determined referring to Chen’s study (Chen et al., 2017). For further western blot detection, HMCs exposed to 30 μg/ml LPS were treated with 20 μM LY294002 (Selleck Chemicals, Shanghai, China), 10 nM insulin-like growth factor 1 (IGF-1, PeproTech, NJ, USA) with or without 40 μM paeoniflorin, for 24 h. Every experiment *in vitro* was repeated at least three times.

### Cell Viability Assay

HMCs (8×10^4^/well) were seeded in 96-well plates until they reached 70-80% confluence, then were treated as described above. After treatments, MTT solution (Biosharp, Hefei, China) at 0.5 mg/ml was added in each well, and the cells were then incubated at 37°C for 4 h. Afterwards, MTT-formazan crystals were solubilized by 150 μl DMSO at room temperature, then the absorbance was measured at 570 nm using a microplate reader (Thermo Fisher Scientific, USA) after thorough mixing by shaking. Cell viability experiment was expressed in “%”, that is the percentage of absorbance in respect to the control (100%).

### Edu Assay

KeyFlour 488 Click EdU kit (KeyGEN, Nanjing, China) was used to detect cell proliferation. HMCs were cultured in 96-well plates and treated as described above. After being fixed with 4% paraformaldehyde for 15 min, cells were incubated with 2 mg/ml glycine for 5 min, then washed twice with 3% BSA/PBS (v/v) and permeabilized with 0.5% Triton-X 100 for 20 min at room temperature. Subsequently, HMCs were incubated with Edu solution at 10 μM for 30 min, then stained with DAPI (Sigma-Aldrich, MO, USA) at 5 μg/ml for 5 min. The images were captured with 20× objective under a fluorescence microscope (DMI3000B, Leica, Wetzlar, Hessen, Germany). The rate of Edu-positive cell (%) was assessed by Image-Pro Plus 6.0 software.

### Cell Cycle Analysis

HMCs were cultured in 12-well plates (2×10^5^/well) and treated as described above. After 24 h treatment, cells were fixed in 70% ice-cold ethanol/PBS (v/v) overnight at 4 °C. Afterwards, HMCs were centrifuged at 800 rpm for 5 min at 4°C and washed with ice-cold PBS twice. After that, HMCs were centrifuged again and resuspended with 500 μl Propidium iodide (PI) staining solution at 50 μg/ml (KeyGEN, Nanjing, China). Samples were then moved to 5-ml BD-Falcon tubes and stored on ice. Flow cytometry analysis was performed by FACS Calibur flow cytometer (Becton Dickinson, SanJose, USA). ModFIT 5.0, a cell cycle analysis software, was used to define the percentage of G0/G1, S and G2/M phase.

### RT-PCR Analysis

RNA of HMCs cultured in 6-well plates (4×10^5^/well) after the same treatments above was prepared using RNAiso Plus (Takara, Beijing, China). Reverse transcription was performed using Prime Script RT Reagent Kit with gDNA Eraser (Takara, Beijing, China) according to the manufacturer’s instruction.

RT-PCR was performed as previously described (Wu et al., 2016), using a detection system (Bio-Rad, Berkeley, CA, USA) with SYBR^®^ Premix Ex TaqTM Ⅱ qPCR Kit (Takara, Beijing China). The relative levels of IL- 1α, IL-2, IL-10 and IFN-γ mRNAs were normalized to GAPDH and calculated according to the 2^-ΔΔCt^ formula. The sequences of the RT-PCR primers were designed by using the Primer Premier 5.0 software (Premier Biosoft, USA) and are shown in **Table 1**.

**Table 1:** Nucleotide sequences of the primers used for qRT-PCR.

Genes	Sense primers (5’’to3’’)	Antisense primers (5’’to3’’)
IL-1α	AGGCTGCATGGATCAATCTGTGTC	CTTCCTCTGAGTCATTGGCGATGG
IL-2	TCCCAAACTCACCAGGATGC	TTGCTGATTAAGTCCCTGGGT
IL-10	GCCAAGCCTTGTCTGAGATGATCC	GCTCCACGGCCTTGCTCTTG
IFN-γ	GTGATGGCTGAACTGTCGCC	ACTGGGATGCTCTTCGACCTC
GAPDH	CAACGTGTCAGTGGTGGACCTG	GTGTCGCTGTTGAAGTCAGAGGAG

### Western Blot Analysis

The proteins of renal cortex tissue and HMCs were extracted and quantified as described previously (Liu et al., 2018). Proteins were loaded and separated in 10% SDS-PAGE, then transferred to polyvinylidene difluoride membranes (Millipore, MA, USA) using a Trans-Blot (Bio-rad, Berkeley, CA, USA). Afterwards, the membranes were blocked with 5% non-fat milk powder (w/v) for 1 h at room temperature. The membranes were incubated with primary antibodies of PI3K (#4257), p-AKT (#4060), AKT (#4691), p-GSK-3β (#5558), GSK-3β (#12456) and Cyclin D1(#2922), which were obtained from Cell Signaling Technology (Danvers, MA, USA) and GAPDH (Lot. IM001-0712, Excell Bio, Shanghai, China) at 4°C overnight. After being washed three times with TBST (Boster, Wuhan, China), the membranes were then incubated with goat anti-rabbit antibody conjugated with horseradish peroxidase at room temperature for 1 h. Finally, these membranes were washed again and detected with an ECL kit (Bio-rad, Berkeley, CA, USA) under a multi-functional imaging system (Tanon 5200, Shanghai, China). All the protein bands were analyzed with Image J 1.48 software.

### Statistical Analysis

Data were presented as mean ± SD. Statistical analysis was performed with the SPSS 18.0 software (Chicago, IL, USA). Except for the results of the 24 h urinary protein, which was analyzed by two-way ANOVA, comparisons among groups were made by one-way ANOVA, followed by Duncan’s test. *P* values less than 0.05 were recognized as significant. Protein bands for western blot were normalized using appropriate controls as mentioned in figure legends.

## Results

### Paeoniflorin Ameliorates Renal Damage in MPGN Model Rats

**Figure 1:**
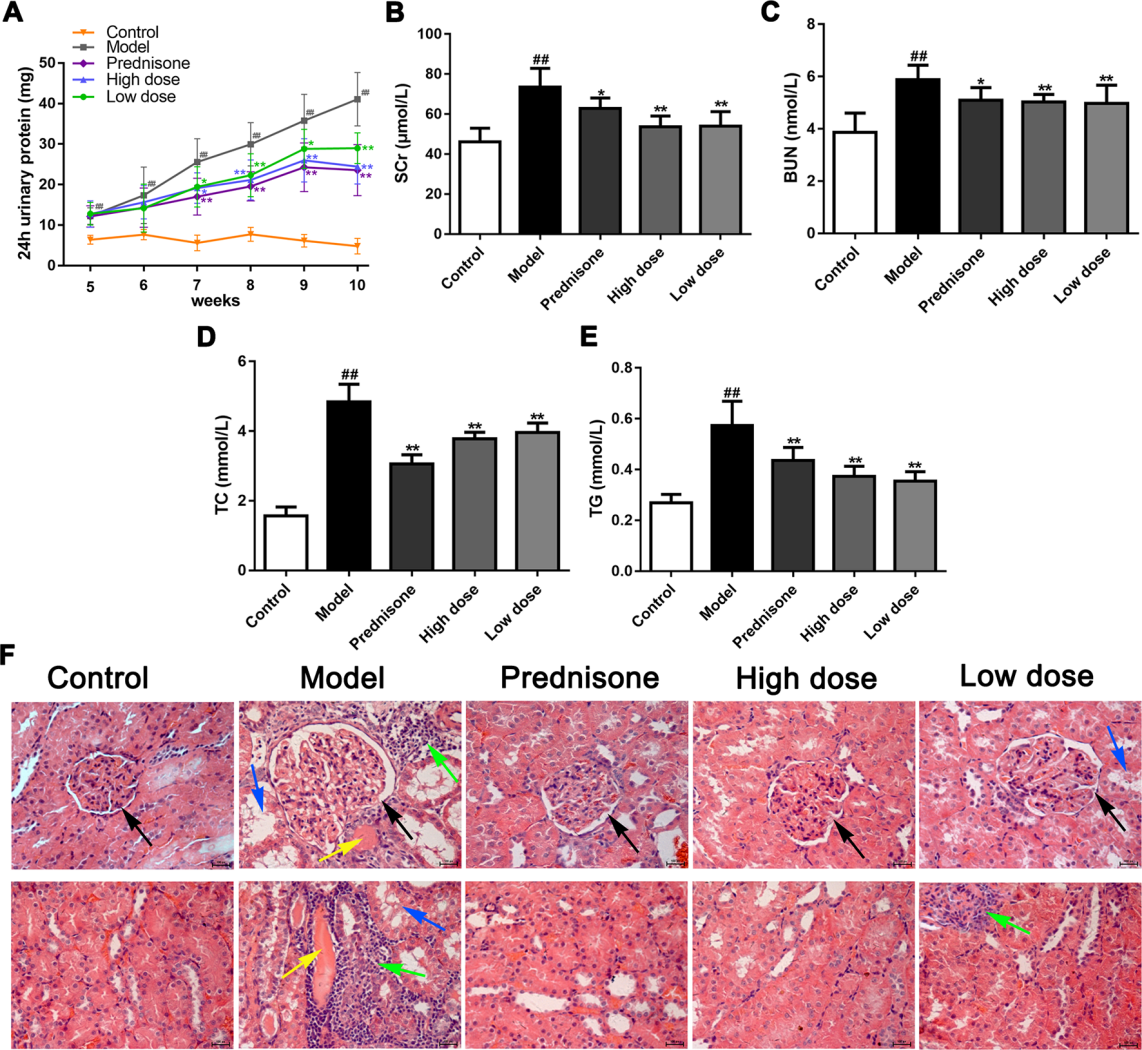
Paeoniflorin ameliorates renal damage in MPGN model rats. Except for eight rats as control group, SD rats (n = 8 per group) were injected BSA and LPS for 5 weeks to establish MPGN model, and then administrated with normal saline (model group), paeoniflorin (50 mg/kg, 100 mg/kg body weight) and prednisone (5 mg/kg body weight) for 5 weeks. **(A)** The content of 24h urinary protein from the 5th week to the 10th week (n = 8). **(B**–**E)** The levels of SCr, BUN, TC and TG in serum (n = 8). **(F)** Kidney tissue sections were obtained and stained with HE (n = 4). Images exhibited glomerulus and tubules were obtained under a light microscope (400× magnification) (scale bar = 20 μm). Black arrows for glomerular structures, green arrows for inflammation infiltration, blue arrows for tubule dilation and yellow arrows for protein casts. Data were expressed as mean ± SD. ^#^ *P* < 0.05 and ^##^ *P* < 0.01 versus control group, *P < 0.05 and **P < 0.01 versus model group.

To confirm the renoprotective effects of paeoniflorin in MPGN model rats, we detected 24 h urinary protein, the levels of Scr, BUN, TC, TG in serum, as well as the renal pathology changes in different groups. Our results showed that paeoniflorin decreased 24 h urinary protein since the seventh week till the end of the animal experiment (**Figure 1A**). Paeoniflorin also reduced the levels of SCr and BUN, exhibiting an improvement of renal function (**Figures 1B, C**). Besides, paeoniflorin lessened the contents of TC and TG, which manifested that paeoniflorin played a protective role in dyslipidemia (**Figures 1D, E**). Furthermore, paeoniflorin decreased the numbers of mesangial cells, improved glomerular structures (black arrows), reduced inflammatory infiltration (green arrows), tubule dilation (blue arrows) and protein cast (yellow arrows) to reverse renal injury (**Figure 1F**). Prednisone, as the positive drug in the animal study, showed protective effects on MPGN, which was similar to paeoniflorin. These results revealed that paeoniflorin ameliorated renal damage in MPGN model rats through protecting against renal function, dyslipidemia and pathological changes.

### Paeoniflorin Inhibits Mesangial Cells Proliferation and Mesangial Matrix Expansion in MPGN Model Rats

**Figure 2:**
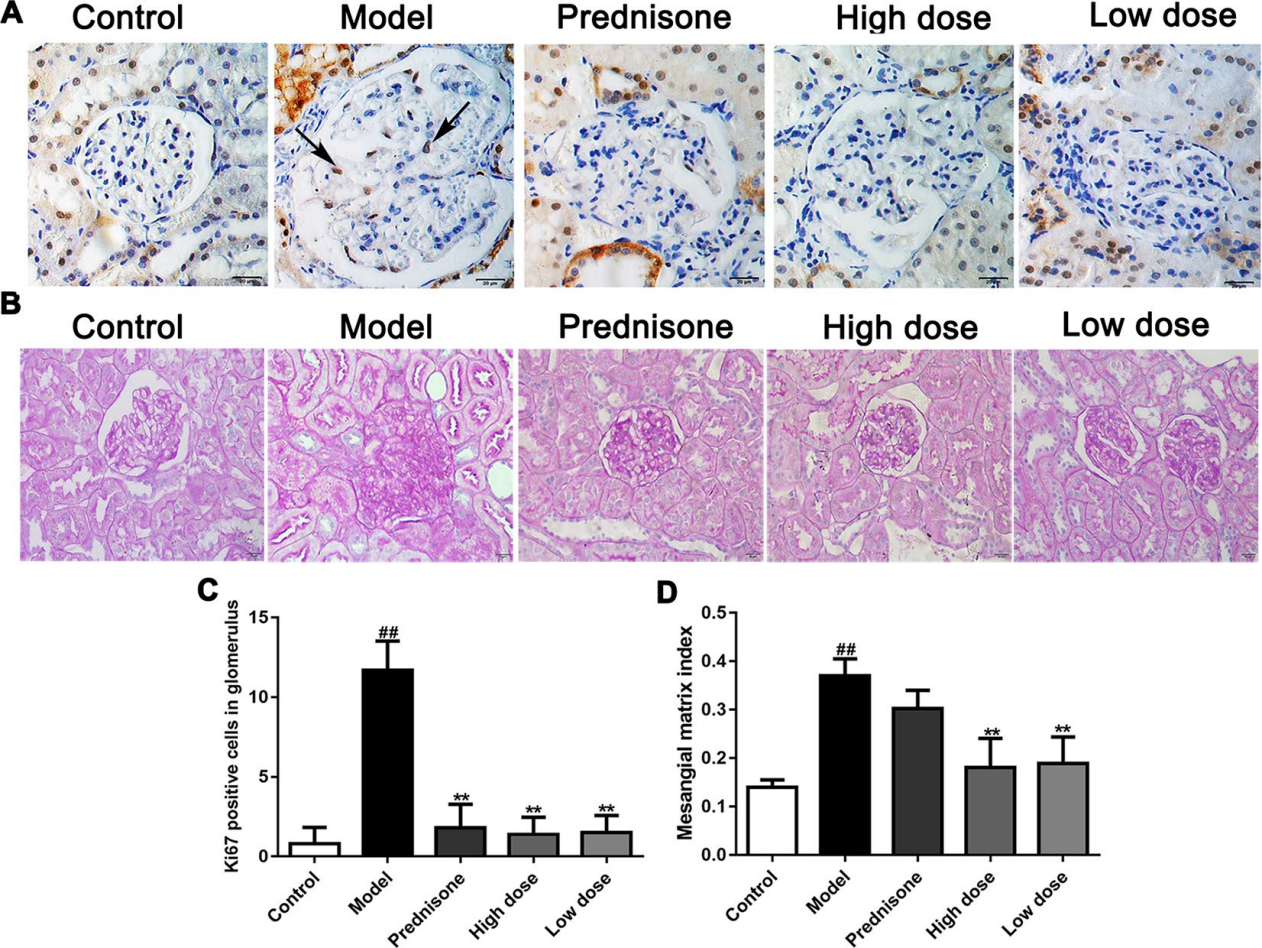
Paeoniflorin suppresses mesangial cell proliferation and mesangial matrix expansion in MPGN model rats. **(A)** The expressions of Ki67 were detected using immunohistochemical staining and indicated by arrows (n = 4). **(B)** Kidney tissue sections were stained with PAS (n = 4). **(C)** Results of Ki67 positive cell number in glomerulus of 10 randomly selected nonoverlapping fields (n = 4). **(D)** Results of mesangial matrix index (n = 20). Images exhibited glomerulus and tubules were obtained under a microscope (400× magnification) (scale bar = 20 μm). Data were expressed as mean ± SD. ^#^ *P* < 0.05 and ^##^ *P* < 0.01 versus control group, *P < 0.05 and **P < 0.01 versus model group.

As a molecular marker of proliferation, the Ki67-positive cells in the mesangial area were regarded as mesangial cell proliferation (Lu et al., 2016). The immunohistochemical results showed that Ki67 was obviously exhibited in the glomerulus of model rats, while the Ki67 positive cells numbers were reduced markedly in the normal and treatment groups (*P* < 0.01) (**Figures 2A, C**). Except for the increased number of mesangial cells, mesangial matrix expansion is one of the characteristics of MPGN. As shown in **Figures 2B, D**, evident mesangial matrix expansion was visible in the model group, as the mesangial matrix index (MMI) of the model group was significantly elevated in comparison to the normal group (*P* < 0.01). Fortunately, the increase of MMI was suppressed by paeoniflorin compared with the model group (*P* < 0.01). Though prednisone distinctly decreased the numbers of Ki67-positive cells in the glomerulus, there was no statistical difference of MMI between the model group and prednisone group. These results demonstrated that paeoniflorin inhibited mesangial cell proliferation and mesangial matrix expansion in MPGN model rats.

### Paeoniflorin Prevents Inflammatory Response in MPGN Model Rats

**Figure 3:**
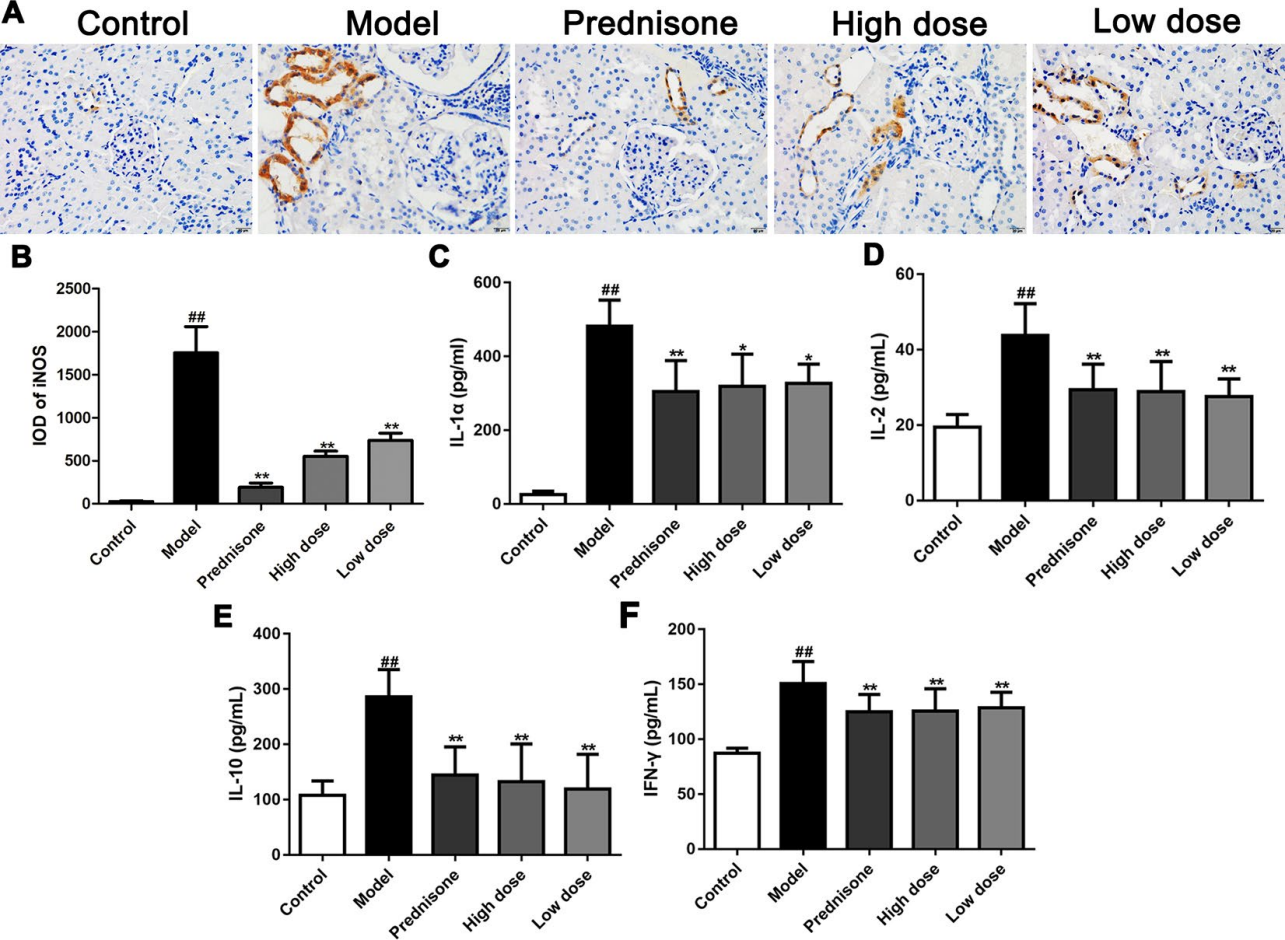
Paeoniflorin prevents inflammatory response in MPGN model rats. **(A)** The expression of iNOS was detected using immunohistochemical staining (n = 4). Brown staining showed positive expression of iNOS. **(B)** Results of integral optical density (IOD) of iNOS (n = 4). **(C**–**F)** The contents of inflammatory factors, IL-1α, IL-2, IL-10 and IFN-γ were measured by using a cytometric bead array kit (n = 8). Images exhibited glomerulus and tubules were obtained under a microscope (400× magnification) (scale bar = 20 μm). Data were expressed as mean ± SD. ^#^ *P* < 0.05 and ^##^ *P* < 0.01 versus control group, *P < 0.05 and **P < 0.01 versus model group.

MPGN, as a typical primary glomerulonephritis, is also accompanied by an increase of inflammatory response. Therefore, immunohistochemistry was performed to detect the expression of iNOS in renal tissues of different groups. As shown in **Figures 3A, B**, both paeoniflorin and prednisone reduced the expression of iNOS in renal tubules (*P* < 0.01). We also carried out a cytometric bead array to measure the levels of IL-1α, IL-2, IL-10 and IFN-γ in serum. Results showed that the treatment of paeoniflorin and prednisone significantly decreased the contents of the above inflammatory cytokines (**Figures 3C–F**). Hence, we can summarize that paeoniflorin presented a good effect on anti-inflammation in MPGN model rats.

### Paeoniflorin Mediates PI3K/AKT/GSK-3β Signaling Pathway in MPGN Model Rats

**Figure 4:**
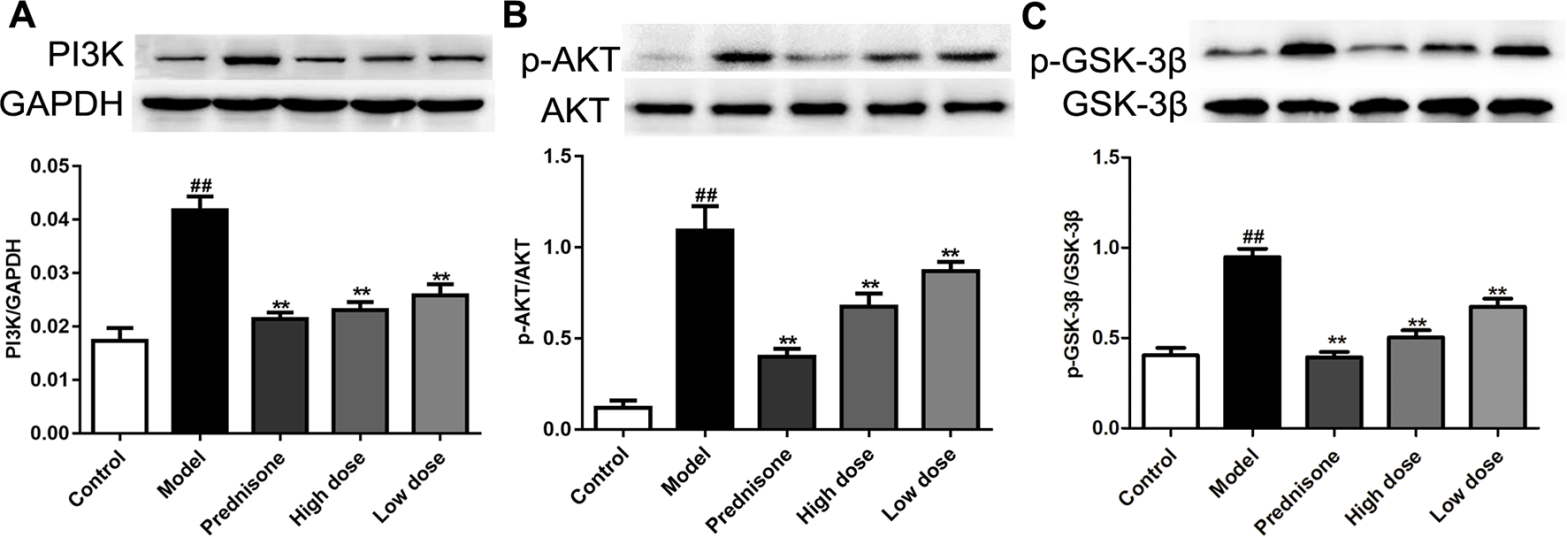
Paeoniflorin mediates the PI3K/AKT/GSK-3β signaling pathway in MPGN model rats. **(A**–**C)** The protein expressions of PI3K, p-AKT, AKT, p-GSK-3β and GSK-3β were analyzed using western blot. The statistical data of all the proteins were analyzed with Image J 1.48 software. The obtained values of PI3K were normalized to the housekeeping gene GAPDH, AKT for p-AKT, and GSK-3β for p-GSK-3β especially. Data were expressed as mean ± SD, n = 4. ^#^ *P* < 0.05 and ^##^ *P* < 0.01 versus control group, *P < 0.05 and **P < 0.01 versus model group.

Recent research reported that enhancing the PI3K/AKT pathway would facilitate mesangial cell proliferation and MPGN (Gao and Han, 2017). Our previous study found that the AKT/GSK-3β pathway is a crucial mechanism of proliferation and inflammatory response in mesangial cells (Wu et al., 2018). In the present study, western blot results showed that the expressions of PI3K, p-AKT and p-GSK-3β are all up-regulated in the model group, which could lead to speculation that the PI3K/AKT/GSK-3β pathway is involved in MPGN pathogenesis (**Figure 4**). On the contrary, paeoniflorin and prednisone notably down-regulated the levels of PI3K, p-AKT and p-GSK-3β (*P* < 0.01). These results revealed that the therapeutic action of paeoniflorin in MPGN by ameliorating mesangial cell proliferation and inflammatory response is probably through regulating the PI3K/AKT/GSK-3β pathway.

### Paeoniflorin Inhibits HMC Proliferation and Affects the Cell Cycle

**Figure 5:**
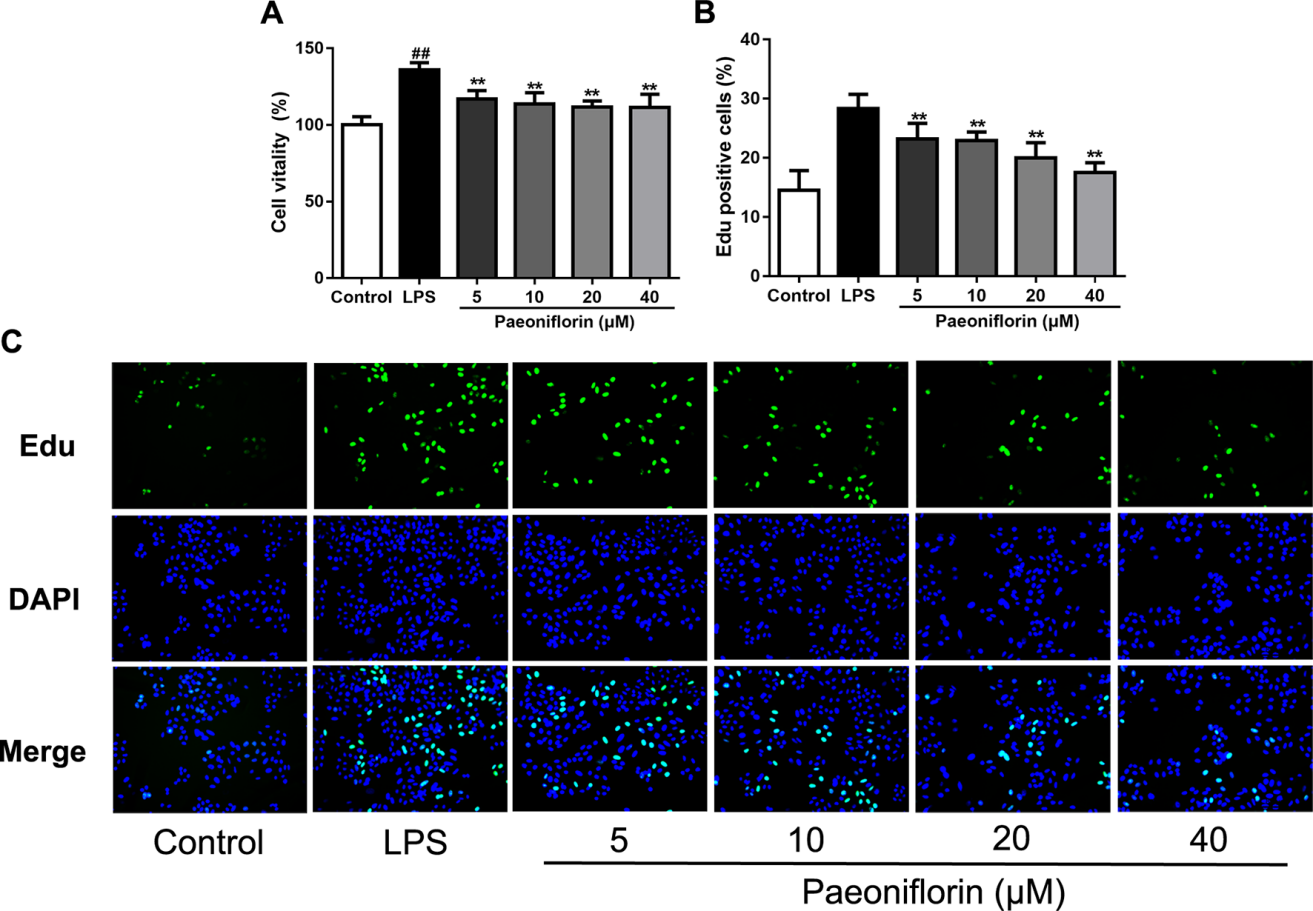
Paeoniflorin inhibits HMC proliferation. HMCs were treated with LPS (30 μg/ml) and incubated with or without paeoniflorin (5, 10, 20, 40 μM) for 24h. **(A)** Cell vitality results of HMCs using MTT assay. The values were expressed in “%”, that is the percentage of absorbance respect to the control (100%) (n = 3). **(B)** The quantification results of Edu-positive cells (%). **(C)** Representative EdU results observed using a light microscope (200× magnification) (scale bar = 20 μm). Data were expressed as mean ± SD, n = 3. ^#^ *P* < 0.05 and ^##^ *P* < 0.01 versus control group, *P < 0.05 and **P < 0.01 versus LPS group.

Both of our previous and recent studies proved that LPS induced HMC proliferation (Sun et al., 2018; Wu et al., 2018). To confirm the role of paeoniflorin in protecting mesangial cells *in vitro*, HMCs were incubated with 30 μg/ml LPS and different dose of paeoniflorin for 24 h. As shown in **Figure 5A**, MTT assay results showed that LPS significantly promoted cell vitality of HMCs (*P* < 0.05), while paeoniflorin (from 5μM to 40μM) inhibited the growth of HMCs (*P* < 0.01). An EdU incorporation assay was used to detect DNA synthesis, which is regarded as an indicator of cell proliferation. The rate of EdU-positive cells was increased in the LPS group, while paeoniflorin obviously reduced EdU incorporation into HMCs (**Figures 5B, C**). Since EdU is a cell proliferation marker that incorporates into S-phase proliferating cells, these data suggest that paeoniflorin may suppress HMC cell proliferation by affecting the cell cycle.

**Figure 6:**
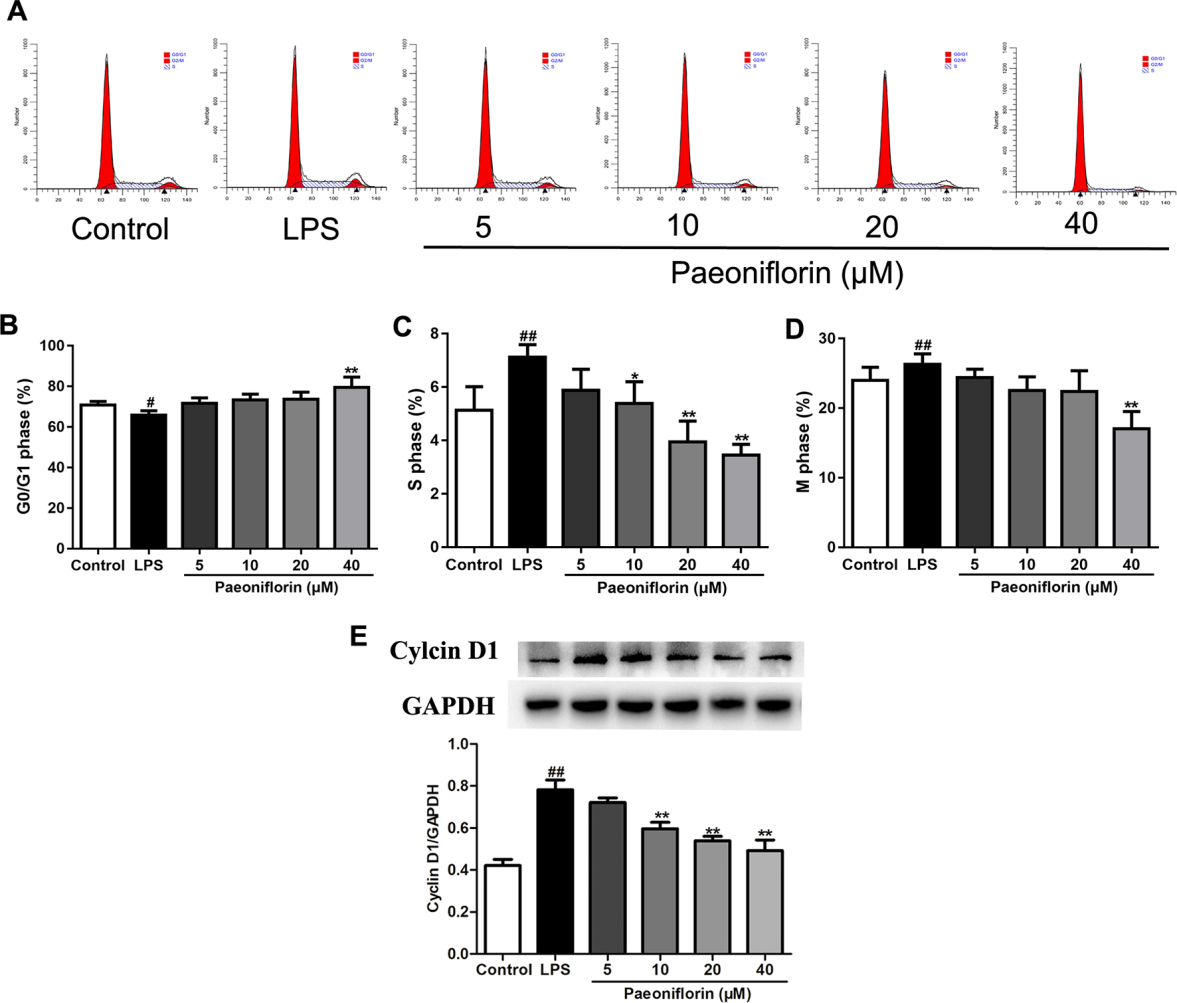
Paeoniflorin affects cell cycle of HMCs. HMC was stained with PI for flow cytometry analysis. **(A)** Representative images of cell cycle. **(B**–**D)** The rate of G0/G1 phage, S phage and M phage (%). **(E)** The protein expressions of Cyclin D1 were analyzed using western blot. The statistical data of the proteins were analyzed with Image J 1.48 software. The obtained values of Cyclin D1 were normalized to the housekeeping gene GAPDH. Data were expressed as mean ± SD, n = 3. ^#^ *P* < 0.05 and ^##^ *P* < 0.01 versus control group, *P < 0.05 and **P < 0.01 versus LPSgroup.

To further evaluate the effect of paeoniflorin on HMC proliferation, we next performed flow cytometry for cell cycle analysis. As shown in **Figures 6A–D**, LPS reduced the percentage of cells in the G0/G1 phase but increased that in the S and G2/M phases, showing that LPS could promote cell cycle progression. Conversely, paeoniflorin at a concentration of 40μM increased the proportion of cells in G1 and decreased that in the S and G2/M phases. These results showed that paeoniflorin could block LPS-induced cell cycle progression by inhibiting G1-S phase transition and arresting cells in G0/G1.

To confirm whether paeoniflorin arrested HMCs on the G1 phase, we also detected the expression of Cyclin D1 by western blot. Cyclin D1 is a key gene in cell cycle regulation which controls the G1-S transition during the cell cycle. A high activity of Cyclin D1 would cause premature cell passage through the G1 checkpoint, which leads to accumulation of DNA damage and finally abnormal cell proliferation (Verim et al., 2013). Our results showed that LPS induced the expression of Cyclin D1, while paeoniflorin decreased it (**Figure 6E**). The results indicated that LPS promoted HMC proliferation, and paeoniflorin inhibited G1-S phase transition and arrested cells in the G0/G1 phase.

### Paeoniflorin Reduces the mRNA Expressions of Inflammatory Factors in HMCs

**Figure 7:**
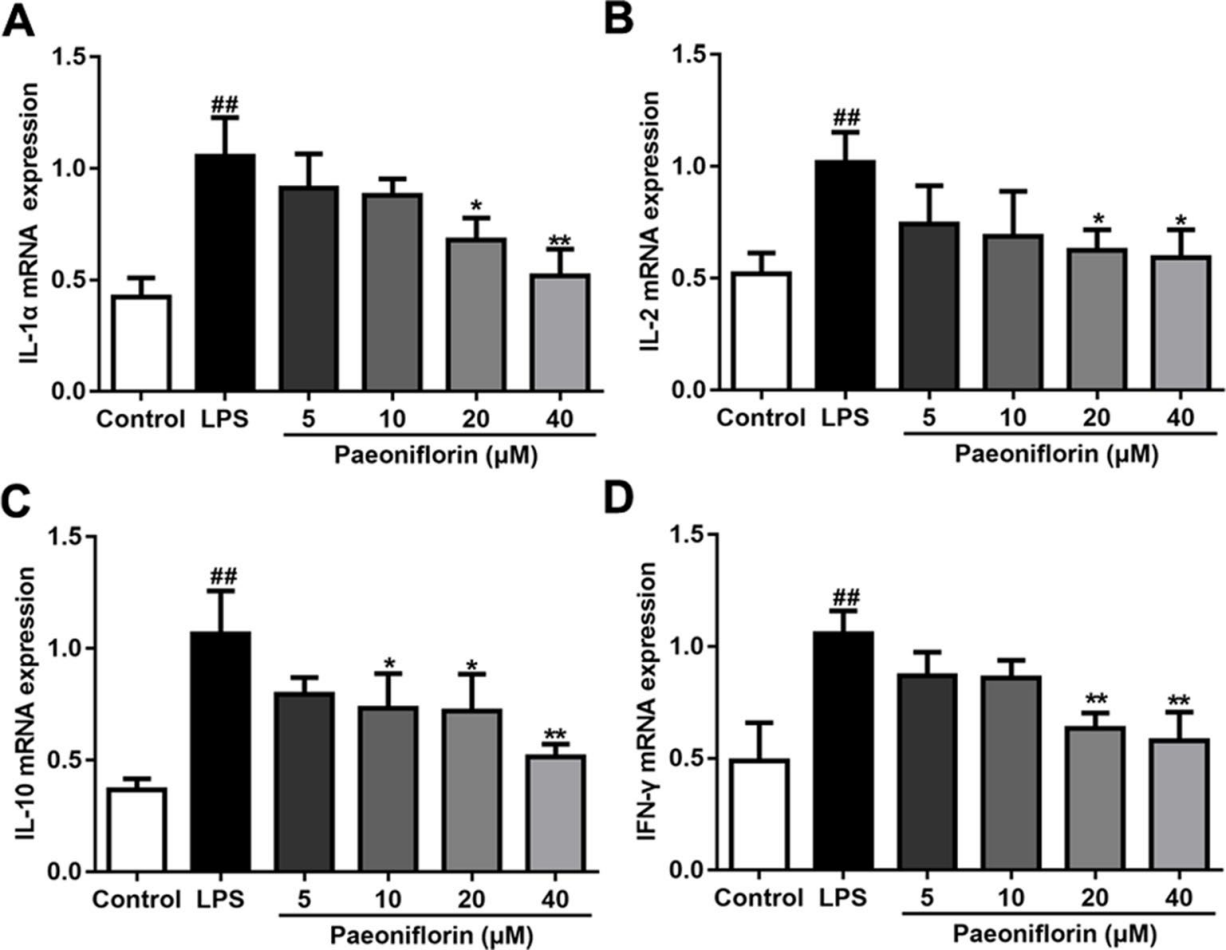
Paeoniflorin reduces the mRNA expressions of inflammatory factors in HMCs. RT-PCR assay was used to determine the mRNA levels of IL-1α, IL-2, IL-10 and IFN-γ. **(A**–**D)** The mRNA expressions of inflammatory factors, IL-1α, IL-2, IL-10 and IFN-γ. Data were expressed as mean ± SD, n = 3. ^#^ *P* < 0.05 and ^##^ *P* < 0.01 versus control group, *P < 0.05 and **P < 0.01 versus LPS group.

LPS also induced inflammatory response in HMCs (Tsugawa et al., 2017), which is in accordance with the rat model of MPGN. To clarify the anti-inflammatory effects of paeoniflorin on HMCs, the mRNA expressions of IL-1α, IL-2, IL-10 and IFN-γ were determined by RT-PCR. As shown in **Figure 7**, paeoniflorin at concentrations of 20 and 40 μM significantly reduced the up-regulation of the examined cytokines induced by LPS (*P* < 0.05 or *P* < 0.01). These results demonstrated that paeoniflorin could inhibit the LPS-mediated inflammatory response of HMCs.

### Paeoniflorin Mediates PI3K/AKT/GSK-3β Signaling Pathway in HMCs

**Figure 8:**
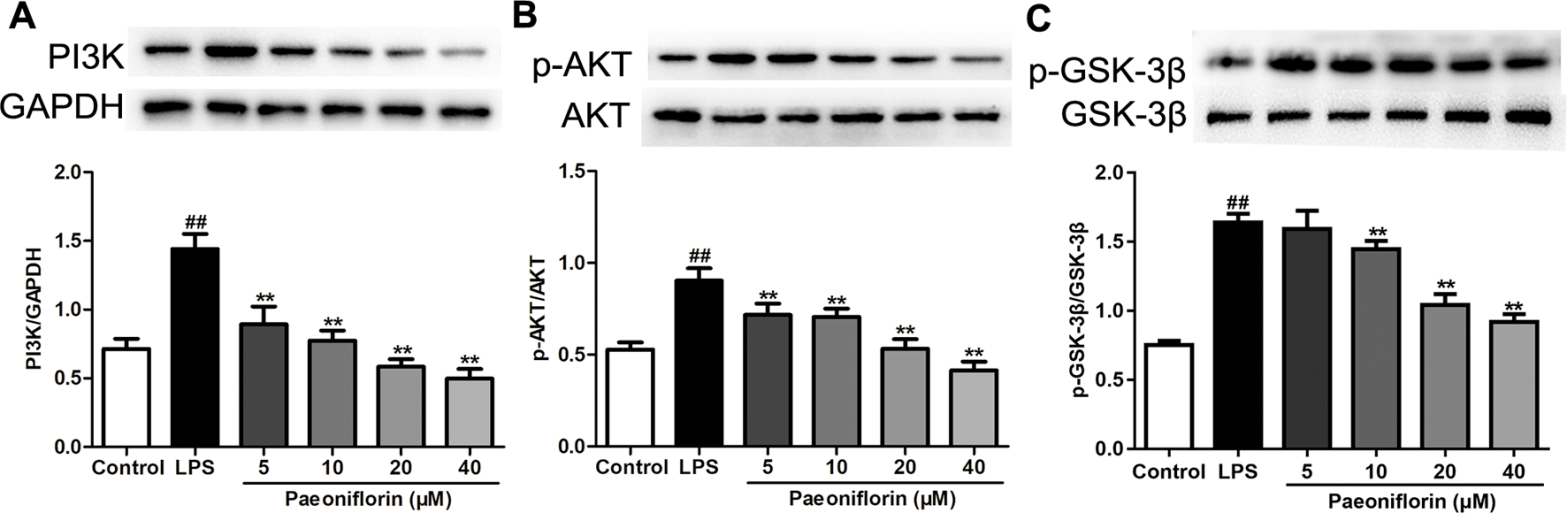
Paeoniflorin mediates the PI3K/AKT/GSK-3β signaling pathway in HMCs. **(A**–**C)** The protein expressions of PI3K, p-AKT, AKT, p-GSK-3β and GSK-3β were analyzed using western blot. The statistical data of all the proteins were analyzed with Image J 1.48 software. The obtained values of PI3K were normalized to the housekeeping gene GAPDH, AKT for p-AKT, and GSK-3β for p-GSK-3β especially. Data were expressed as mean ± SD, n = 3. ^#^ *P* < 0.05 and ^##^ *P* < 0.01 versus control group, *P < 0.05 and **P < 0.01 versus LPS group.

To confirm the molecular mechanisms underlying the anti-proliferative and anti-inflammatory effects of paeoniflorin against HMCs induced by LPS, the protein expressions of the PI3K/AKT/GSK-3β pathway were determined. As shown in **Figure 8**, the protein expressions of PI3K, p-AKT and p-GSK-3β were increased in the LPS group (*P* < 0.01). Luckily, paeoniflorin significantly suppressed the expressions of PI3K, p-AKT and p-GSK-3β (*P* < 0.01), showing that paeoniflorin possibly prevented HMC injury through regulating the PI3K/AKT/GSK-3β pathway.

**Figure 9:**
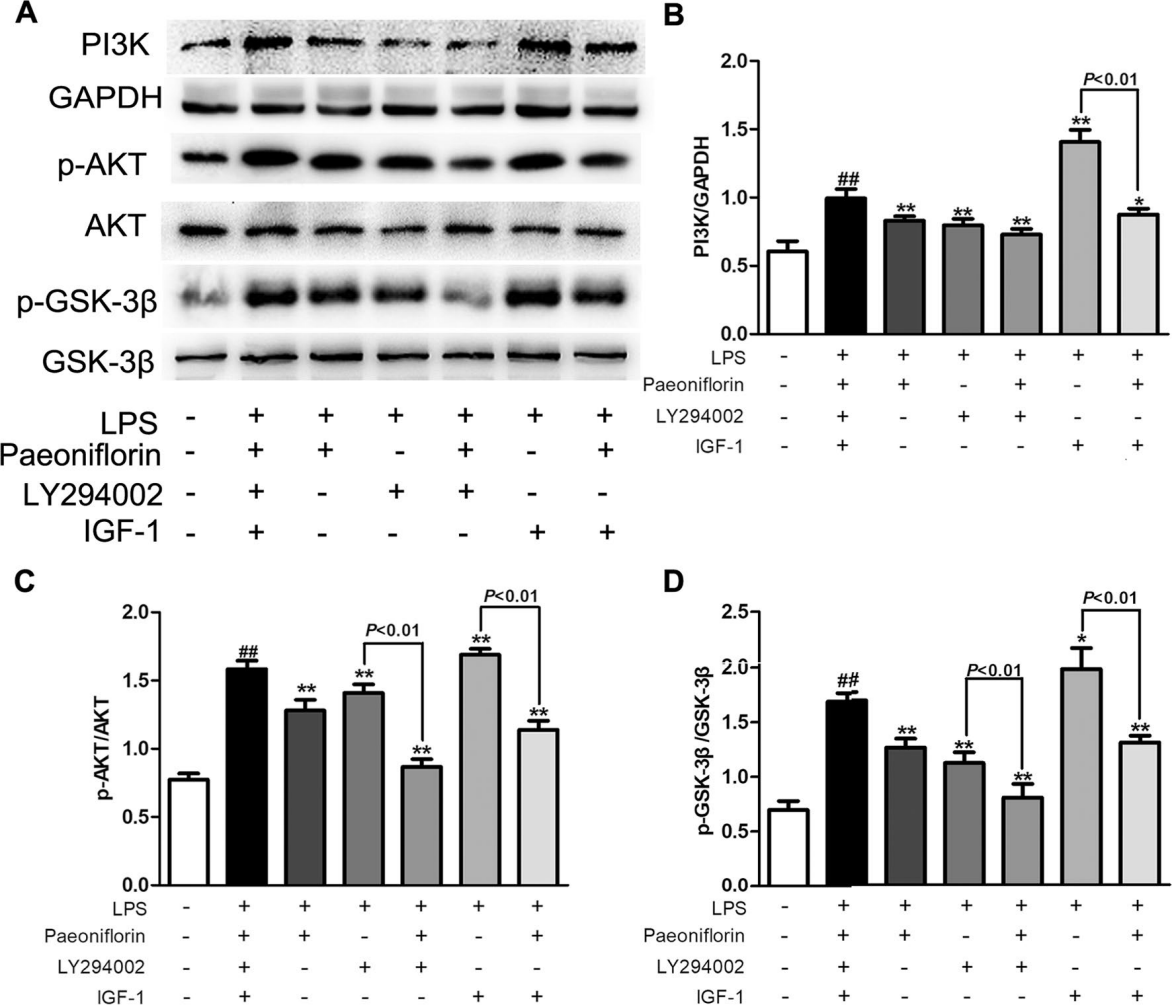
The effects of LY204002 and IGF-1 on HMC proliferation in response to paeoniflorin. HMCs exposed to LPS (30 μg/ml) were treated with LY294002 (20 μM), IGF (10 nM) with or without paeoniflorin (40 μM) for 24h. **(A)** The protein expressions of PI3K, p-AKT, AKT, p-GSK-3β and GSK-3β were analyzed using western blot. **(B**–**D)** The statistical data of all the proteins were analyzed with Image J 1.48 software. The obtained values of PI3K were normalized to the housekeeping gene GAPDH, AKT for p-AKT, and GSK-3β for p-GSK-3β especially. Data were expressed as mean ± SD, n = 3. ^#^ *P* < 0.05 and ^##^ *P* < 0.01 versus control group, *P < 0.05 and **P < 0.01 versus LPS group.

PI3K is a crucial molecule in regulating the function of mesangial cells, targeting the activation of AKT/GSK-3β signaling in kidneys. To further investigate whether the protective effects of paeoniflorin were mediated by the PI3K/AKT/GSK-3β pathway, we detected the effects of paeoniflorin under the intervention of PI3K inhibitor LY294002, or PI3K agonist IGF-1 in HMCs. As shown in **Figure 9**, the protein levels of PI3K and its downstream proteins p-AKT and p-GSK-3β were inhibited by LY294002 and increased by IGF-1, compared to the LPS group (*P* < 0.01 or *P* < 0.05). Paeoniflorin further suppressed the phosphorylation of AKT and GSK-3β when combined application with LY294002. Remarkably, paeoniflorin reversed the suppression of the PI3K/AKT/GSK-3β signaling pathway induced by IGF-1, which demonstrated the definite inhibition of paeoniflorin on the PI3K.

## Discussion

Paeoniflorin is one of the active compounds in *Paenia radix* Alba, which is an important herb used in traditional Chinese medicine to treat chronic kidney disease or other chronic inflammatory diseases (Li et al., 2017). Our previous study found that paeoniflorin ameliorated nephrotic syndrome model rats through inhibiting inflammation and podocyte apoptosis (Lu et al., 2017). Some studies reported that paeoniflorin improved oxidative damage and inflammation in mesangial cells (Chen et al., 2017; Zhang et al., 2013). Mesangial cells are the most active intrinsic cells in the glomerulus, while proliferative reaction is their most common response to several stimulations, such as immune complexes, hypoxia and macromolecular substances. MPGN is characterized by mesangial cell proliferation and mesangial matrix expansion. Therefore, suppression of mesangial cell proliferation would be the most important way to treat MPGN. However, whether paeoniflorin could be a potential drug in MPGN still unclear.

In the present study, we observed the protective effects of paeoniflorin on MPGN and explored the underlying mechanism. For this purpose, a MPGN rat model established by BSA and LPS was used, which was also known as chronic serum sickness (CSS), characterized by glomerular immune complex deposition, hypercellularity and matrix expansion (Bao et al., 2007). Our data demonstrated that paeoniflorin lessened the number of mesangial cells, inhibited mesangial matrix expansion and reduced the expressions of Ki67 in MPGN. We also carried out a HMCs proliferation model induced by LPS to evaluate the effects of paeoniflorin *in vitro*. Results showed that paeoniflorin suppressed cell viability and reduced proliferative cell staining with Edu. In addition, paeoniflorin blocked cell cycle progression in HMCs induced by LPS and down-regulated the expression of Cyclin D1. These results suggested that paeoniflorin has potent effects against mesangial cells proliferation *in vivo* and *in vitro*.

The activated and proliferative mesangial cells in the glomerulus could produce kinds of vasoactive substances and cytokines including interleukin-1 (IL-1), interferon (IFN) and chemotactic factor (CXC), thereby promoting ongoing mesangial cell proliferation (Gao et al., 2017). In fact, inflammation plays a key role in the development of MPGN, including anti-Thy1 nephritis and IgA nephropathy animal models (Hua et al., 2013; Aizawa et al., 2014). Our results showed that the levels of inflammatory factors, IL-1α, IL-2, IL-10 and IFN-γ were augmented in MPGN model rats, as well as in HMCs treated with LPS. Moreover, the expressions of macrophage marker iNOS were increased in tubules in MPGN model rats. Paeoniflorin reversed the inflammatory response by lessening the levels of inflammatory factors and the expressions of iNOS. In consequence, our results demonstrated that paeoniflorin inhibited inflammatory response in MPGN.

Among the three classes of PI3Ks, class IA PI3Ks have been studied most extensively. Class IA PI3Ks are heterodimeric enzymes composed of a regulatory p85 subunit and a p110 catalytic subunit (Chiu et al., 2014). An increase of p85 could stabilize p110 proteins which would augment p85–p110 heterodimers, thereby enhancing PI3K activity (Kok et al., 2009). Moreover, the increased levels of p85 upon cellular stimulation correlates with sustained AKT phosphorylation in cells (Esposito et al., 2008). Recent reports showed that the PI3K/AKT/GSK-3β pathway has been reported involved in cell proliferation (Chan et al., 2018), apoptosis (Xie et al., 2017), and inflammation (Jing et al., 2017) in various kidney diseases. Currently accumulating evidence supports that the PI3K/AKT signaling pathway contributes to the promotion of proliferation in mesangial cells (Feng et al., 2016; Ying et al., 2017). Additionally, one study reported that phosphorylation of AKT is involved in the initiation and development of mesangial proliferation in anti-Thy1 MPGN model rats (Qiu et al., 2012). GSK-3β, a major downstream signaling molecule of AKT participating in various cellular processes, plays a crucial role in inflammation and proliferation (Hong et al., 2017; Xie et al., 2018). More importantly, GSK-3 inhibitors may emerge as effective drugs for proliferative kidney diseases (Soos et al., 2006). Recent research reported that paeoniflorin inhibits cell proliferation through suppression of PI3K/AKT signaling (Wu et al., 2013; Zheng et al., 2015). In the present study, the PI3K/AKT/GSK-3β signaling pathway was activated in the MPGN model rats and HMCs induced by LPS, as the expressions of PI3K, p-AKT, p-GSK-3β were markedly increased. After paeoniflorin treatment, the expressions of related proteins were decreased. These results illustrated that paeoniflorin improves MPGN mostly through regulating the PI3K/AKT/GSK-3β pathway.

Similarly, the effects of paeoniflorin on reducing inflammatory cytokines (IL-1α, IL-2, IL-10, IFNγ) may be related to the inhibition of the PI3K/AKT/GSK-3β signaling pathway. It was reported that the PI3K inhibitor LY294002 reduced the level of IL-2 in T lymphocytes through inhibiting the PI3K/AKT/GSK-3β pathway (Braunstein et al., 2008). Methane-rich saline induced the contents of IL-10 and IFN-γ through activating the PI3K/AKT/GSK-3β pathway (Yao et al., 2017). However, the molecular mechanism underlying regulating of anti-inflammatory cytokines of the PI3K pathway in our study is poorly understood. Research illustrated that GSK-3β can activate the transcriptional activity of NF-κB by phosphorylation of its p65 subunit (Chen et al., 2018; Yang et al., 2019). Inhibition of GSK-3β affects macrophage polarization and inhibits NF-kB activation, and that results in potent anti-inflammatory effects in several animal models (Liu et al., 2016; Suber et al., 2017; Tantray et al., 2017; Wang et al., 2018). Meanwhile, GSK-3β gene deletion prevents cell differentiation induced by NF-κB (Hoeflich et al., 2000). GSK-3β inhibitor lithium chloride and SB415286 leads to the reduction of pro-inflammatory molecules by decreasing NF-κB activity (Wang et al., 2013). Furthermore, research showed that LPS could induce cell proliferation and the contents of inflammatory cytokines of HMCs through activation of NF-κB nuclear translocation (Jiang et al., 2011). Therefore, it is reasonable to presume that the anti-inflammatory effects of paeoniflorin on mesangial cells and MPGN are related to the NF-κB pathway by suppressing GSK-3β.

LY294002 is a kind of PI3K inhibitor that can inhibit the activity of PI3K and AKT and inactivate GSK-3β (Song et al., 2018). Insulin-like growth factor-1 (IGF-1) is known to bind to the IGF-1 receptor as well as the PI3K agonist (Li et al., 2015). To confirm the regulation of paeoniflorin on the PI3K/AKT/GSK-3β pathway, LY294002 and IGF-1 were administrated alone or combined with paeoniflorin in HMCs. Fortunately, we found that paeoniflorin enhanced the inhibitory effects on the activation of the PI3K/AKT/GSK-3β pathway by LY294002. On the other hand, paeoniflorin reversed the effects of IGF-1. These results manifested that paeoniflorin protects HMC injury mostly through down-regulating PI3K.

In conclusion, paeoniflorin displayed a positive therapeutic effect on MPGN *in vivo* and *in vitro* by inhibiting mesangial cells proliferation and inflammation response. More importantly, we first demonstrated the potential mechanisms of effective prevention of paeoniflorin on MPGN, namely by down-regulating the PI3K/AKT/GSK-3β signaling pathway. Indeed, paeoniflorin may be a novel therapeutic agent for MPGN.

## Ethics Statement

This study was carried out in accordance with the recommendations of Animal Ethics Committee of Guangzhou University of Chinese Medicine. The protocol was approved by Animal Ethics Committee of Guangzhou University of Chinese Medicine

## Author Contributions

JZ designed the experiments. BL, JL, LB and RL performed *in vivo* research. PZ, DC and HL performed *in vitro* research. JS, XL and YW contributed to the preparation of reagents, materials and experimental equipment. CL and JW analyzed the data. BL and YZ wrote the manuscript. All authors read and approved the final manuscript.

## Funding

This research was supported by the National Natural Science Foundation of China (no.81673874, and 81803824), the National Natural Science Foundation of Guangdong Province (no.2016A030310292, 2018A030313328 and 2018B0303110004), the Education Department of Guangdong Province (no.2016KZDXM030), the PhD Start-up Fund of the Natural Science Foundation of Guangdong Province (no.2017A030310127) and the Medical Scientific Research Foundation of Guangdong Province (no.A2017144).

## Conflict of Interest Statement

The authors declare that the research was conducted in the absence of any commercial or financial relationships that could be construed as a potential conflict of interest.

## References

[B1] Aizawa K. Tashiro Y. Hirata M. Takeda S. Kawasaki R. Endo K. (2014). Renoprotective effect of epoetin beta pegol by the prevention of M2 macrophage recruitment in Thy-1 rats. J. Nephrol. 27 (4), 395–401. 10.1007/s40620-014-0099-3 24821659

[B2] Bai J. Geng W. Mei Y. Wu L. Duan S. Dong Z. (2017). Effect of huaier on the proliferation of mesangial cells in anti-thy-1 nephritis. Cell Physiol. Biochem. 42 (6), 2441–2452. 10.1159/000480198 28848114

[B3] Bao L. Haas M. Minto A. W. Quigg R. J. (2007). Decay-accelerating factor but not CD59 limits experimental immune-complex glomerulonephritis. Lab. Invest. 87 (4), 357–364. 10.1038/labinvest.3700522 17259999

[B4] Braunstein J. Autschbach F. Giese T. Lasitschka F. Heidtmann A. Sido B. (2008). Up-regulation of the phosphoinositide 3-kinase pathway in human lamina propria T lymphocytes. Clin. Exp. Immunol. 151 (3), 496–504. 10.1111/j.1365-2249.2007.03562.x 18234058 PMC2276955

[B5] Chan K. K. L. Siu M. K. Y. Jiang Y. X. Wang J. J. Leung T. H. Y. Ngan H. Y. S. (2018). Estrogen receptor modulators genistein, daidzein and ERB-041 inhibit cell migration, invasion, proliferation and sphere formation *via* modulation of FAK and PI3K/AKT signaling in ovarian cancer. Cancer Cell Int. 18, 65. 10.1186/s12935-018-0559-2 29743815 PMC5930957

[B6] Chen C. L. Cheng M. H. Kuo C. F. Cheng Y. L. Li M. H. Chang C. P. (2018). Dextromethorphan attenuates NADPH oxidase-regulated glycogen synthase kinase 3beta and NF-kappaB activation and reduces nitric oxide production in group a streptococcal infection. Antimicrob. Agents Chemother. 62 (6). 10.1128/AAC.02045-17 PMC5971618 29581121

[B7] Chen J. Zhao D. Zhu M. Zhang M. Hou X. Ding W. (2017). Paeoniflorin ameliorates AGEs-induced mesangial cell injury through inhibiting RAGE/mTOR/autophagy pathway. Biomed. Pharmacother. 89, 1362–1369. 10.1016/j.biopha.2017.03.016 28320103

[B8] Chiu Y. H. Lee J. Y. Cantley L. C. (2014). BRD7, a tumor suppressor, interacts with p85alpha and regulates PI3K activity. Mol. Cell 54 (1), 193–202. 10.1016/j.molcel.2014.02.016 24657164 PMC4004185

[B9] Esposito I. Proto M. C. Gazzerro P. Laezza C. Miele C. Alberobello A. T. (2008). The cannabinoid CB1 receptor antagonist rimonabant stimulates 2-deoxyglucose uptake in skeletal muscle cells by regulating the expression of phosphatidylinositol-3-kinase. Mol. Pharmacol. 74 (6), 1678–1686. 10.1124/mol.108.049205 18801918

[B10] Feng X. Wu C. Yang M. Liu Q. Li H. Liu J. (2016). Role of PI3K/Akt signal pathway on proliferation of mesangial cell induced by HMGB1. Tissue Cell 48 (2), 121–125. 10.1016/j.tice.2015.12.007 26822343

[B11] Fruman D. A. Chiu H. Hopkins B. D. Bagrodia S. Cantley L. C. Abraham R. T. (2017). The PI3K pathway in human disease. Cell 170 (4), 605–635. 10.1016/j.cell.2017.07.029 28802037 PMC5726441

[B12] Gao H. Y. Han C. X. (2017). The role of PTEN up-regulation in suppressing glomerular mesangial cells proliferation and nephritis pathogenesis. Eur. Rev. Med. Pharmacol. Sci. 21 (16), 3634–3641. 10.26355/eurrev_201708_13276 28925480

[B13] Gao J. Wu L. Wang Y. Cui S. Duan S. Dong Z. (2017). Knockdown of Cxcl10 Inhibits mesangial cell proliferation in murine habu nephritis *via* ERK signaling. Cell Physiol. Biochem. 42 (5), 2118–2129. 10.1159/000479914 28810249

[B14] Geng W. Wei R. Liu S. Tang L. Zhu H. Chen P. (2016). Shenhua Tablet inhibits mesangial cell proliferation in rats with chronic anti-Thy-1 nephritis. Biol. Res. 49, 17. 10.1186/s40659-016-0078-3 26969153 PMC4788853

[B15] Gu X. Cai Z. Cai M. Liu K. Liu D. Zhang Q. (2016). Protective effect of paeoniflorin on inflammation and apoptosis in the cerebral cortex of a transgenic mouse model of Alzheimer’s disease. Mol. Med. Rep. 13 (3), 2247–2252. 10.3892/mmr.2016.4805 26796245

[B16] Hoeflich K. P. Luo J. Rubie E. A. Tsao M. S. Jin O. Woodgett J. R. (2000). Requirement for glycogen synthase kinase-3beta in cell survival and NF-kappaB activation. Nature 406 (6791), 86–90. 10.1038/35017574 10894547

[B17] Hogendoorn P. C. Bruijn J. A. Gelok E. W. Van den Broek L. J. Fleuren G. J. (1990). Development of progressive glomerulosclerosis in experimental chronic serum sickness. Nephrol. Dial. Transplant. 5 (2), 100–109. 10.1093/ndt/5.2.100 2141388

[B18] Hong H. Chen F. Qiao Y. Yan Y. Zhang R. Zhu Z. (2017). GSK-3beta activation index is a potential indicator for recurrent inflammation of chronic rhinosinusitis without nasal polyps. J. Cell Mol. Med. 21 (12), 3633–3640. 10.1111/jcmm.13274 28714566 PMC5706567

[B19] Hua K. F. Yang S. M. Kao T. Y. Chang J. M. Chen H. L. Tsai Y. J. (2013). Osthole mitigates progressive IgA nephropathy by inhibiting reactive oxygen species generation and NF-kappaB/NLRP3 pathway. PLoS One 8 (10), e77794. 10.1371/journal.pone.0077794 24204969 PMC3810132

[B20] Jiang Q. Liu P. Wu X. Liu W. Shen X. Lan T. (2011). Berberine attenuates lipopolysaccharide-induced extracelluar matrix accumulation and inflammation in rat mesangial cells: involvement of NF-kappaB signaling pathway. Mol. Cell Endocrinol. 331 (1), 34–40. 10.1016/j.mce.2010.07.023 20674665

[B21] Jing D. Bai H. Yin S. (2017). Renoprotective effects of emodin against diabetic nephropathy in rat models are mediated *via* PI3K/Akt/GSK-3beta and Bax/caspase-3 signaling pathways. Exp. Ther. Med. 14 (5), 5163–5169. 10.3892/etm.2017.5131 29201232 PMC5704304

[B22] Kok K. Geering B. Vanhaesebroeck B. (2009). Regulation of phosphoinositide 3-kinase expression in health and disease. Trends Biochem. Sci. 34 (3), 115–127. 10.1016/j.tibs.2009.01.003 19299143

[B23] Li C. Zhang J. Ma Z. Zhang F. Yu W. (2018). miR-19b serves as a prognostic biomarker of breast cancer and promotes tumor progression through PI3K/AKT signaling pathway. Onco. Targets Ther. 11, 4087–4095. 10.2147/OTT.S171043 30038508 PMC6052917

[B24] Li H. Jiao Y. Xie M. (2017). paeoniflorin ameliorates atherosclerosis by suppressing TLR4-mediated NF-kappaB activation. Inflammation 40 (6), 2042–2051. 10.1007/s10753-017-0644-z 28791506

[B25] Li H. Xu L. Zhao L. Ma Y. Zhu Z. Liu Y. (2015). Insulin-like growth factor-I induces epithelial to mesenchymal transition *via* GSK-3beta and ZEB2 in the BGC-823 gastric cancer cell line. Oncol. Lett. 9 (1), 143–148. 10.3892/ol.2014.2687 25435948 PMC4246767

[B26] Liu B. He Y. Lu R. Zhou J. Bai L. Zhang P. (2018). Zhen-wu-tang protects against podocyte injury in rats with IgA nephropathy *via* PPARgamma/NF-kappaB pathway. Biomed. Pharmacother. 101, 635–647. 10.1016/j.biopha.2018.02.127 29518610

[B27] Liu X. Liu C. Li J. Zhang X. Song F. Xu J. (2016). Urocortin attenuates myocardial fibrosis in diabetic rats *via the* Akt/GSK-3beta signaling pathway. Endocr. Res. 41 (2), 148–157. 10.3109/07435800.2015.1094489 26934363

[B28] Lu R. Zhou J. Liu B. Liang N. He Y. Bai L. (2017). Paeoniflorin ameliorates Adriamycin-induced nephrotic syndrome through the PPARgamma/ANGPTL4 pathway *in vivo* and *in vitro* . Biomed. Pharmacother. 96, 137–147. 10.1016/j.biopha.2017.09.105 28972886

[B29] Lu Y. Wen J. Chen D. Wu L. Li Q. Xie Y. (2016). Modulation of cyclins and p53 in mesangial cell proliferation and apoptosis during Habu nephritis. Clin. Exp. Nephrol. 20 (2), 178–186. 10.1007/s10157-015-1163-6 26359229 PMC4819602

[B30] Ma X. H. Duan W. J. Mo Y. S. Chen J. L. Li S. Zhao W. (2018). Neuroprotective effect of paeoniflorin on okadaic acid-induced tau hyperphosphorylation *via* calpain/Akt/GSK-3beta pathway in SH-SY5Y cells. Brain Res. 1690, 1–11. 10.1016/j.brainres.2018.03.022 29596798

[B31] Ma Z. Liu H. Wang W. Guan S. Yi J. Chu L. (2017). Paeoniflorin suppresses lipid accumulation and alleviates insulin resistance by regulating the Rho kinase/IRS-1 pathway in palmitate-induced HepG2Cells. Biomed. Pharmacother. 90, 361–367. 10.1016/j.biopha.2017.03.087 28380411

[B32] Qin D. Morita H. Inui K. Tayama H. Inoue Y. Yoshimura A. (2013). Aldosterone mediates glomerular inflammation in experimental mesangial proliferative glomerulonephritis. J. Nephrol. 26 (1), 199–206. 10.5301/jn.5000125 22641568

[B33] Qiu W. Zhang Y. Liu X. Zhou J. Li Y. Zhou Y. (2012). Sublytic C5b-9 complexes induce proliferative changes of glomerular mesangial cells in rat Thy-1 nephritis through TRAF6-mediated PI3K-dependent Akt1 activation. J. Pathol. 226 (4), 619–632. 10.1002/path.3011 21984198

[B34] Song L. Dong X. Zhu S. Zhang C. Yin W. Zhang X. (2018). Bi2 S3 -Tween 20 nanodots loading PI3K inhibitor, LY294002, for mild photothermal therapy of oVo cells *In Vitro* and *In Vivo* . Adv. Healthc Mater. 7 (22), e1800830. 10.1002/adhm.201800830 30240165

[B35] Song S. Xiao X. Guo D. Mo L. Bu C. Ye W. (2017). Protective effects of Paeoniflorin against AOPP-induced oxidative injury in HUVECs by blocking the ROS-HIF-1alpha/VEGF pathway. Phytomedicine 34, 115–126. 10.1016/j.phymed.2017.08.010 28899493

[B36] Soos T. J. Meijer L. Nelson P. J. (2006). CDK/GSK-3 inhibitors as a new approach for the treatment of proliferative renal diseases. Drug News Perspect. 19 (6), 325–328. 10.1358/dnp.2006.19.6.985939 16971968

[B37] Suber T. Wei J. Jacko A. M. Nikolli I. Zhao Y. Zhao J. (2017). SCF(FBXO17) E3 ligase modulates inflammation by regulating proteasomal degradation of glycogen synthase kinase-3beta in lung epithelia. J. Biol. Chem. 292 (18), 7452–7461. 10.1074/jbc.M116.771667 28298444 PMC5418045

[B38] Sun P. F. Tian T. Chen L. N. Fu R. G. Xu S. S. Ai H. (2018). ultrasound combined with microbubbles enhances the effects of methylprednisolone in lipopolysaccharide-induced human mesangial cells. J. Pharmacol. Exp. Ther. 365 (3), 476–484. 10.1124/jpet.117.246223 29549156

[B39] Sun X. Li S. Xu L. Wang H. Ma Z. Fu Q. (2017). Paeoniflorin ameliorates cognitive dysfunction *via* regulating SOCS2/IRS-1 pathway in diabetic rats. Physiol. Behav. 174, 162–169. 10.1016/j.physbeh.2017.03.020 28322909

[B40] Tantray M. A. Khan I. Hamid H. Alam M. S. Dhulap A. Ganai A. A. (2017). Oxazolo[4,5-b]pyridine-based piperazinamides as GSK-3beta inhibitors with potential for attenuating inflammation and suppression of pro-inflammatory mediators. Arch. Pharm. (Weinheim) 350 (8), e1700022. 10.1002/ardp.201700022 28543747

[B41] Tsugawa K. Imaizumi T. Watanabe S. Tsuruga K. Yoshida H. Tanaka H. (2017). Clarithromycin attenuates the expression of monocyte chemoattractant protein-1 by activating toll-like receptor 4 in human mesangial cells. Clin. Exp. Nephrol. 21 (4), 573–578. 10.1007/s10157-016-1333-1 27614743

[B42] Verim A. Ozkan N. Turan S. Korkmaz G. Cacina C. Yaylim I. (2013). Association of the Cylin D1 G870A polymorphism with laryngeal cancer: are they really related? Asian Pac. J. Cancer Prev. 14 (12), 7629–7634. 10.7314/APJCP.2013.14.12.7629 24460344

[B43] Wang C. D. Yuan C. F. Bu Y. Q. Wu X. M. Wan J. Y. Zhang L. (2014). Fangchinoline inhibits cell proliferation *via* Akt/GSK-3beta/cyclin D1 signaling and induces apoptosis in MDA-MB-231 breast cancer cells. Asian Pac. J. Cancer Prev. 15 (2), 769–773. 10.7314/APJCP.2014.15.2.769 24568493

[B44] Wang C. Y. Tsai A. C. Peng C. Y. Chang Y. L. Lee K. H. Teng C. M. (2012). Dehydrocostuslactone suppresses angiogenesis *in vitro* and *in vivo* through inhibition of Akt/GSK-3beta and mTOR signaling pathways. PLoS One 7 (2), e31195. 10.1371/journal.pone.0031195 22359572 PMC3281050

[B45] Wang H. M. Zhang T. Li Q. Huang J. K. Chen R. F. Sun X. J. (2013). Inhibition of glycogen synthase kinase-3beta by lithium chloride suppresses 6-hydroxydopamine-induced inflammatory response in primary cultured astrocytes. Neurochem. Int. 63 (5), 345–353. 10.1016/j.neuint.2013.07.003 23871716

[B46] Wang L. Wang Y. Zhang C. Li J. Meng Y. Dou M. (2018). inhibiting glycogen synthase kinase 3 reverses obesity-induced white adipose tissue inflammation by regulating apoptosis inhibitor of macrophage/CD5L-mediated macrophage migration. Arterioscler Thromb. Vasc. Biol. 38 (9), 2103–2116. 10.1161/ATVBAHA.118.311363 30026270

[B47] Wang P. Wang W. Shi Q. Zhao L. Mei F. Li C. (2016). Paeoniflorin ameliorates acute necrotizing pancreatitis and pancreatitisinduced acute renal injury. Mol. Med. Rep. 14 (2), 1123–1131. 10.3892/mmr.2016.5351 27279569 PMC4940107

[B48] Wu J. He Y. Luo Y. Zhang L. Lin H. Liu X. (2018). MiR-145-5p inhibits proliferation and inflammatory responses of RMC through regulating AKT/GSK pathway by targeting CXCL16. J. Cell Physiol. 233 (4), 3648–3659. 10.1002/jcp.26228 29030988

[B49] Wu J. Liu B. Liang C. Ouyang H. Lin J. Zhong Y. (2016). Zhen-wu-tang attenuates cationic bovine serum albumin-induced inflammatory response in membranous glomerulonephritis rat through inhibiting AGEs/RAGE/NF-kappaB pathway activation. Int. Immunopharmacol. 33, 33–41. 10.1016/j.intimp.2016.01.008 26851631

[B50] Wu Y. M. Jin R. Yang L. Zhang J. Yang Q. Guo Y. Y. (2013). Phosphatidylinositol 3 kinase/protein kinase B is responsible for the protection of paeoniflorin upon H(2)O(2)-induced neural progenitor cell injury. Neuroscience 240, 54–62. 10.1016/j.neuroscience.2013.02.037 23485815

[B51] Xie W. Lu J. Lu Q. Wang X. Long H. Huang J. (2018). Matrine inhibits the proliferation and migration of lung cancer cells through regulation of the protein kinase B/glycogen synthase kinase-3beta signaling pathways. Exp. Ther. Med. 16 (2), 723–729. 10.3892/etm.2018.6266 30112033 PMC6090456

[B52] Xie X. C. Zhao N. Xu Q. H. Yang X. Xia W. K. Chen Q. (2017). Relaxin attenuates aristolochic acid induced human tubular epithelial cell apoptosis *in vitro* by activation of the PI3K/Akt signaling pathway. Apoptosis 22 (6), 769–776. 10.1007/s10495-017-1369-z 28386751

[B53] Yang S. Zhang X. Qu H. Qu B. Yin X. Zhao H. (2019). Cabozantinib induces PUMA-dependent apoptosis in colon cancer cells *via* AKT/GSK-3beta/NF-kappaB signaling pathway. Cancer Gene. Ther. 10.1038/s41417-019-0098-6 PMC7237347 31182761

[B54] Yang Y. Zhang Z. Zhuo L. Chen D. P. Li W. G. (2018). The spectrum of biopsy-proven glomerular disease in china: a systematic review. Chin. Med. J. (Engl) 131 (6), 731–735. 10.4103/0366-6999.226906 29521297 PMC5865320

[B55] Yao Y. Wang L. Jin P. Li N. Meng Y. Wang C. (2017). Methane alleviates carbon tetrachloride induced liver injury in mice: anti-inflammatory action demonstrated by increased PI3K/Akt/GSK-3beta-mediated IL-10 expression. J. Mol. Histol. 48 (4), 301–310. 10.1007/s10735-017-9728-1 28597201

[B56] Ying C. Chen L. Wang S. Mao Y. Ling H. Li W. (2017). Zeaxanthin ameliorates high glucose-induced mesangial cell apoptosis through inhibiting oxidative stress *via* activating AKT signalling-pathway. Biomed. Pharmacother. 90, 796–805. 10.1016/j.biopha.2017.04.013 28431381

[B57] Zeng J. Dou Y. Guo J. Wu X. Dai Y. (2013). Paeoniflorin of Paeonia lactiflora prevents renal interstitial fibrosis induced by unilateral ureteral obstruction in mice. Phytomedicine 20 (8–9), 753–759. 10.1016/j.phymed.2013.02.010 23535189

[B58] Zhai T. Sun Y. Li H. Zhang J. Huo R. Li H. (2016). Unique immunomodulatory effect of paeoniflorin on type I and II macrophages activities. J. Pharmacol. Sci. 130 (3), 143–150. 10.1016/j.jphs.2015.12.007 26852260

[B59] Zhang H. Li F. Pan Z. Wu Z. Wang Y. Cui Y. (2014). Activation of PI3K/Akt pathway limits JNK-mediated apoptosis during EV71 infection. Virus Res. 192, 74–84. 10.1016/j.virusres.2014.07.026 25116390

[B60] Zhang J. Yu K. Han X. Zhen L. Liu M. Zhang X. (2018). Paeoniflorin influences breast cancer cell proliferation and invasion *via* inhibition of the Notch1 signaling pathway. Mol. Med. Rep. 17 (1), 1321–1325. 10.3892/mmr.2017.8002 29115554

[B61] Zhang M. H. Feng L. Zhu M. M. Gu J. F. Wu C. Jia X. B. (2013). Antioxidative and anti-inflammatory activities of paeoniflorin and oxypaeoniflora on AGEs-induced mesangial cell damage. Planta Med. 79 (14), 1319–1323. 10.1055/s-0033-1350649 23881455

[B62] Zhang T. Zhu Q. Shao Y. Wang K. Wu Y. (2017). Paeoniflorin prevents TLR2/4-mediated inflammation in type 2 diabetic nephropathy. Biosci. Trends 11 (3), 308–318. 10.5582/bst.2017.01104 28626209

[B63] Zheng Y. B. Xiao G. C. Tong S. L. Ding Y. Wang Q. S. Li S. B. (2015). Paeoniflorin inhibits human gastric carcinoma cell proliferation through up-regulation of microRNA-124 and suppression of PI3K/Akt and STAT3 signaling. World J. Gastroenterol. 21 (23), 7197–7207. 10.3748/wjg.v21.i23.7197 26109806 PMC4476881

